# Knockdown of lncRNA MIR31HG inhibits cell proliferation in human HaCaT keratinocytes

**DOI:** 10.1186/s40659-018-0181-8

**Published:** 2018-09-04

**Authors:** Jintao Gao, Fangru Chen, Mingchun Hua, Junfan Guo, Yuejuan Nong, Qinyan Tang, Fengxia Zhong, Linxiu Qin

**Affiliations:** 1 College of Biotechnology, Guilin Medical University, Guilin, 541100 Guangxi People’s Republic of China; 2 Department of Dermatology, Affiliated Hospital of Guilin Medical University, Guilin, 541001 Guangxi People’s Republic of China; 3 Department of Plastic Surgery, Affiliated Hospital of Guilin Medical University, Guilin, 541001 Guangxi People’s Republic of China

**Keywords:** Knockdown, LncRNA, MIR31HG, Proliferation, HaCaT keratinocytes

## Abstract

**Background:**

Psoriasis is a complex, chronic inflammatory skin disease with substantial negative effects on patient quality of life. Long non-coding RNAs (lncRNAs) are able to be involved in multitudes of cellular processes in diverse human diseases. This study aimed to investigate the potential involvement of lncRNA MIR31HG in HaCaT keratinocytes proliferation.

**Results:**

The study showed that MIR31HG was significantly elevated in the lesional psoriatic skin compared with normal individuals’ skin. Knockdown of MIR31HG inhibited HaCaT keratinocytes proliferation. Flow cytometry analysis showed that siRNA-mediated MIR31HG depletion induced cell cycle arrest in the G2/M phase. In addition, MIR31HG expression was found to be dependent on NF-κB activation.

**Conclusions:**

NF-κB activation mediated MIR31HG upregulation plays an important role in the regulation of HaCaT keratinocytes proliferation. It could be a potential diagnostic biomarker and therapeutic target for psoriasis.

## Background

Psoriasis is a complex, chronic inflammatory skin disease affecting 2–3% of the population worldwide [1]. Psoriasis is thought to be initiated by complex interactions between genetic and environmental factors [2]. Psoriasis lesions are characterized by epidermal hyperproliferation and aberrant differentiation of keratinocytes and infiltration of inflammatory cells into the dermis and epidermis, which manifests clinically in the thickening and scaling of skin lesions [3]. However, the underlying mechanisms remain largely elusive.

Long non-coding RNAs (lncRNAs) are defined as transcripts longer than 200 nucleotides, with no protein coding capacity [4]. Increasing evidences suggest that lncRNAs play essential roles in a wide range of biological processes [5]. Multiple lines of evidence link dysregulations of lncRNAs to diverse human diseases [6]. LncRNA PRINS (Psoriasis susceptibility-related RNA Gene Induced by Stress) have been found to play important roles in the proliferation of keratinocytes and may contribute to psoriasis susceptibility [7–9]. LncRNA-MSX2P1 acts as a ceRNA (competitive endogenous RNA) directly binding to miR-6731-5p and regulating the expression of S100A7, thus affecting the development of psoriasis [10]. MIR31HG (also known as LOC554202), the host gene of intronic miRNA miR-31 is a 2166-nt lncRNA. MIR31HG has been reported to affect cell proliferation in various cancers [11–13]. However, the function of MIR31HG in psoriasis has not been investigated.

RNA-seq data revealed that MIR31HG is elevated in psoriasis lesions versus normal controls [14], suggesting MIR31HG may play a potential role in the pathogenesis of psoriasis. In the present study, we aimed to investigate the expression of MIR31HG in psoriasis lesions and normal skins and determine the effects of MIR31HG knockdown on the proliferation of human HaCaT keratinocytes.

## Results

### MIR31HG is upregulated in psoriasis lesions

**Fig. 1:**
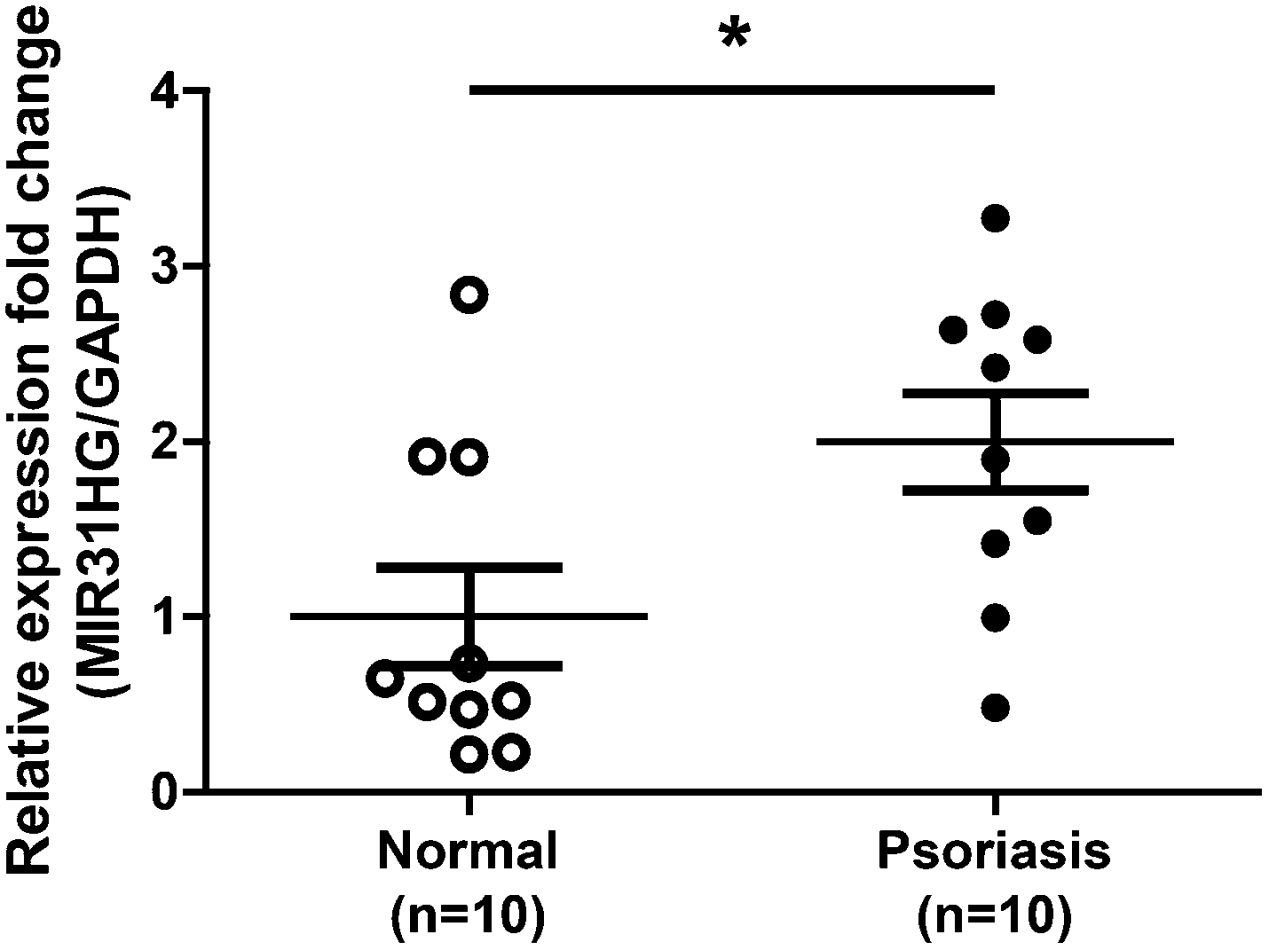
MIR31HG expression is elevated in psoriatic skin. qRT-PCR was performed to measure the relative expression fold change of MIR31HG in the psoriatic skin and normal individuals’ skin tissues. GAPDH served as an internal reference. *P < 0.05

qRT-PCR assay was used to determine the expression of MIR31HG in the lesional skin tissues from psoriasis patients and normal skin tissues from healthy individuals. The results showed that MIR31HG levels were significantly higher in lesional skin tissues compared with normal tissues (Fig. 1).

### Knockdown of MIR31HG inhibits HaCaT keratinocytes proliferation

**Fig. 2:**
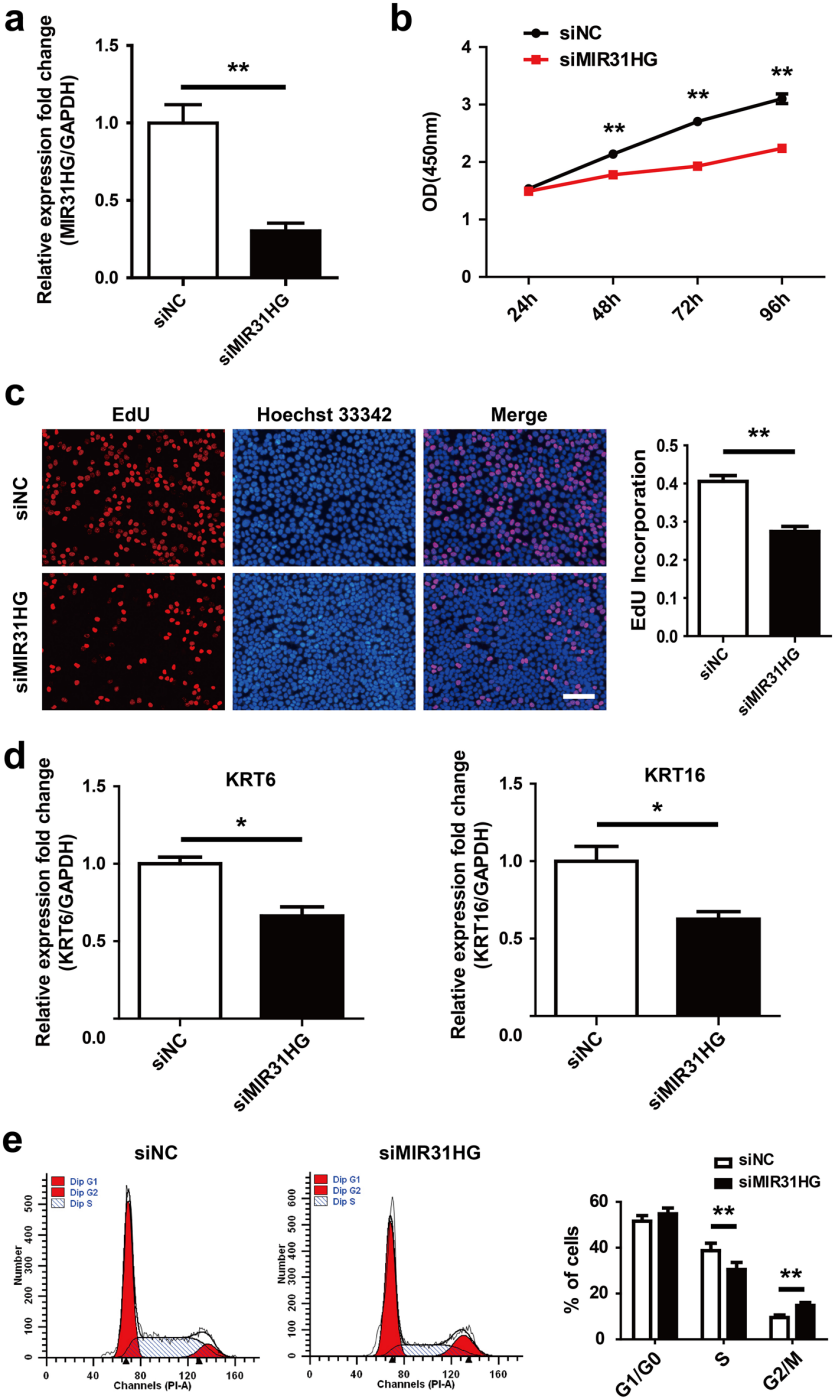
MIR31HG knockdown inhibits HaCaT keratinocytes proliferation. **a** HaCaT keratinocytes were transfected with 50 nM scramble siRNA (siNC) or MIR31HG siRNA (siMIR31HG) for 48 h. qRT-PCR was performed to measure the expression of MIR31HG. Transcript levels were normalized to GAPDH expression. **P < 0.01. **b** CCK-8 assays were carried out to record the growth curves of HaCaT keratinocytes transfected with 50 nM siNC or siMIR31HG. **P < 0.01. **c** EdU incorporation assays were used to detect the proliferation rate of HaCaT keratinocytes transfected with 50 nM siNC or siMIR31HG for 48 h. EdU incorporation rate was analyzed using Image-pro Plus 6.0 software. Scale bars, 100 μm. **P < 0.01. **d** KRT6 and KRT16 expression was measured by qRT-PCR in HaCaT keratinocytes transfected with 50 nM siNC or siMIR31HG for 48 h. *P < 0.05. **e** The cell cycle distribution of HaCaT keratinocytes at 48 h post-transfection with 50 nM siNC or siMIR31HG was analyzed by flow cytometry. **P < 0.01

In order to determine the possible effects of aberrant MIR31HG expression on keratinocyte proliferation, we applied siRNA targeting MIR31HG to suppress its expression in HaCaT keratinocytes. qRT-PCR assay showed that MIR31HG expression was significantly suppressed by specific siRNA (Fig. 2a). The growth curves detected by CCK8 showed that MIR31HG knockdown dramatically decreased HaCaT keratinocytes growth from 48 h post-transfection (Fig. 2b). Consistent with the results of CCK8 assays, EdU incorporation was significantly reduced following siRNA transfection (Fig. 2c). Keratin 6 (KRT6) and keratin 16 (KRT16) are considered to be the hallmarks of psoriatic keratinocytes hyperproliferation [15–17]. qRT-PCR assay showed that MIR31HG knockdown resulted in significant reduction of the expression of KRT6 and KRT16 (Fig. 2d). These results indicated that MIR31HG knockdown can inhibit HaCaT keratinocytes proliferation. In order to explore the potential reasons for cell growth suppression after MIR31HG knockdown, the cell cycle was analyzed by flow cytometry. MIR31HG silencing significantly decreased the percentage of S phase cells and increased the percentage of G2/M phase cells, indicating that MIR31HG knockdown can inhibit HaCaT keratinocytes proliferation by the induction of G2/M arrest (Fig. 2e).

### NF-κB activation is required for the upregulation of MIR31HG

**Fig. 3:**
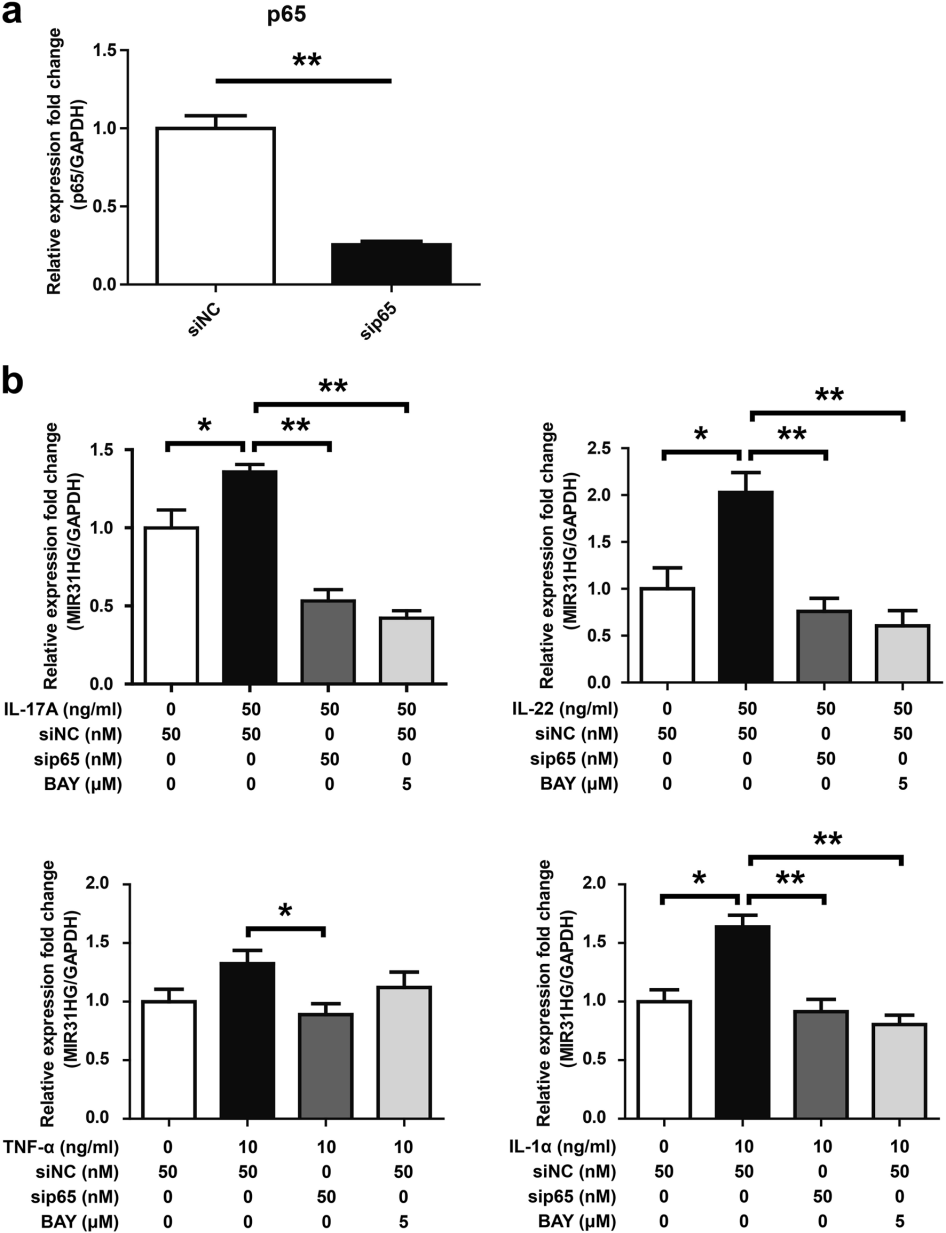
MIR31HG expression is dependent on NF-κB signaling. **a** The relative expression fold change of p65 mRNA was measured by qRT-PCR in HaCaT keratinocytes transfected with 50 nM siNC or sip65 for 48 h. GAPDH served as an internal reference. **P < 0.01. **b** HaCaT keratinocytes transfected with sip65 or pretreated with BAY11-7082 (BAY) for 1 h were stimulated with different kinds of cytokines (IL-17A, IL-22, TNF-α or IL-1α) for 24 h. The relative expression fold change of MIR31HG transcript was measured by qRT-PCR. GAPDH served as an internal reference. *P < 0.05, **P < 0.01

miR-31 is located within the intron of MIR31HG. NF-κB activation is essential for miR-31 expression [18]. In order to investigate whether the expression of MIR31HG is dependent on NF-κB activation, we stimulated HaCaT keratinocytes with IL-17A, IL-22, TNF-α and IL-1α. In psoriasis, pro-inflammatory cytokines including IL-17A, IL-22, TNF-α and IL-1α may contribute to the pathophysiology of the disease [2, 19]. IL-17A, IL-22, TNF-α and IL-1α have been reported to be able to activate NF-κB signaling [20, 21]. We found that all of the tested inflammatory cytokines were able to stimulate MIR31HG expression (Fig. 3b). In addition, specific siRNA targeting p65 was applied to suppress its expression. qRT-PCR assay showed that p65 expression was significantly suppressed by specific siRNA (Fig. 3a). p65 knockdown resulted in significant reduction of the expression of MIR31HG in HaCaT keratinocytes stimulated with different kinds of cytokines (IL-17A, IL-22, TNF-α or IL-1α) (Fig. 3b). Consistent with the results of sip65, disruption of NF-κB pathway by BAY11-7082 (BAY, NF-κB inhibitor) abolished cytokines-induced MIR31HG expression (Fig. 3b). These data indicated that MIR31HG expression is dependent on NF-κB activation.

## Discussion

Psoriasis is a chronic, immune-mediated skin disease characterized by keratinocyte hyperproliferation and immune cell infiltration [1]. Although a lot of details are known regarding its pathogenesis, much remains to be elucidated and understood. LncRNAs are a new class of regulatory RNAs being involved in multitudes of cellular processes [5]. Recently, transcriptome approaches identified distinct lncRNA expression profile in psoriatic lesions [14, 22], suggesting lncRNAs may play potential roles in the pathogenesis of psoriasis.

In this study, we observed that the expression of MIR31HG is elevated in the lesional skin compared with normal individuals’ skin (Fig. 1). MIR31HG has been found dysregulated in human cancers and can regulate the cell proliferation positively or negatively [11, 23]. To better understand the potential roles of MIR31HG during psoriasis, we transfected with MIR31HG-specific siRNA and analyzed the effect on HaCaT keratinocytes proliferation. By MIR31HG-specific siRNA transfection, MIR31HG expression decreased 70% (Fig. 2a), and this MIR31HG silencing resulted in inhibition of HaCaT keratinocytes growth (Fig. 2b). EdU, a nucleoside analog of thymidine, is readily incorporated into cellular DNA during DNA replication. EdU assay showed that the percentage of incorporated cells is reduced following MIR31HG knockdown (Fig. 2c). KRT6/KRT16 keratin pair is induced in hyperproliferative psoriatic keratinocytes, but not found in normal keratinocytes [24]. KRT6 and KRT16 are considered to be hyperproliferation-associated keratins and to be the hallmarks of psoriasis [15–17]. MIR31HG-deficient cells exhibited reduced levels of KRT6 and KRT16 (Fig. 2d). Moreover, flow cytometry analysis showed that siRNA-mediated MIR31HG depletion induced cell cycle arrest in the G2/M phase (Fig. 2e).

Dysregulated crosstalk between dysfunctional keratinocytes and abnormal immune cells may drive keratinocyte hyperproliferation and aberrant differentiation in psoriasis. Keratinocytes are responsive to key dendritic cell-derived and T-cell-derived cytokines including interferons, TNF, IL-17, and the IL-20 family of cytokines, and in turn produce proinflammatory cytokines to sustain or even shape the psoriatic inflammatory process [1]. NF-κB signaling may well act as a link in this crosstalk [3]. miR-31 is located within the intron of MIR31HG, and its induction requires NF-κB activation [18]. In order to investigate whether MIR31HG expression requires NF-κB activation, we stimulated HaCaT keratinocytes with IL-17A, IL-22, TNF-α and IL-1α. These cytokines have been reported to be able to activate NF-κB signaling [20, 21]. Our results showed that IL-17A, IL-22, TNF-α and IL-1α were able to elevate MIR31HG expression (Fig. 3b). In addition, siRNA-mediated knockdown of p65 resulted in significant reduction of cytokines-induced MIR31HG expression (Fig. 3b). The similar results were observed in HaCaT keratinocytes treated with BAY11-7082 (BAY, NF-κB inhibitor) (Fig. 3b). These data indicated that MIR31HG expression is dependent on NF-κB activation. Our data highlight the critical role of NF-κB signaling pathway in psoriatic keratinocyte hyperproliferation. These data suggest that MIR31HG may be an attractive target for therapeutic intervention of psoriasis in the future.

## Conclusions

In conclusion, knockdown of MIR31HG inhibits the proliferation of HaCaT keratinocytes resulted from induction of cell cycle arrest. MIR31HG expression is dependent on NF-κB activation. Our findings provide evidence that MIR31HG plays an important role in regulation of keratinocytes hyperproliferation in psoriasis and may be an attractive therapeutic target. However, further studies are required to clarify the mechanism for MIR31HG knockdown inducing cell cycle arrest in HaCaT keratinocytes.

## Methods

### Cell culture and exposure to IL-17A, IL-22, TNF-α or IL-1α

HaCaT keratinocytes were obtained from Kunming Cell Bank, Kunming Institute of Zoology, Chinese Academy of Science (Kunming, China), cultured in Dulbecco’s modified Eagle’s medium (DMEM) containing 10% Fetal Bovine Serum (FBS) and maintained at 37 °C with 5% CO_2_.

IL-17A, IL-22, TNF-α and IL-1α were purchased from PeproTech (RockyHill, NJ, USA). Cells were cultured to 80% confluence and incubated with IL-17A (50 ng/ml), IL-22 (50 ng/ml), TNF-α (10 ng/ml) or IL-1α (10 ng/ml) for 24 h.

### Human tissue samples

Punch biopsies (4 mm) were taken from lesional skin areas of 10 patients with psoriasis who received no medical treatment within recent 4 weeks (7 male and 3 female, average age 53.3 years). Normal skin specimens were taken from healthy individuals undergoing plastic surgery. All samples were collected immediately, snap frozen in liquid nitrogen and stored at − 80 °C until RNA extraction. Before participation in this study, all individuals provided informed consent. The study was approved by the Ethics Committee of Affiliated Hospital of Guilin Medical University.

### Quantitative RT-PCR assays (qRT-PCR)

Total RNA was isolated from HaCaT keratinocytes and skin biopsies using TRIzol™ reagent (Invitrogen). cDNA was synthesized using RevertAid First Strand cDNA Synthesis Kit (ThermoFisher). Quantitative PCR was carried out with SYBR Green qPCR Master Mix (Biomake, Houston, USA) as described previously [25] in CFX96 Touch™ Real-Time PCR Detection System (Bio-Rad). The reaction procedure was 95 °C for 5 min, followed by 40 cycles at 95 °C for 5 s, 60 °C for 10 s and 72 °C for 10 s. GAPDH served as an internal reference. The relative gene expression was analyzed by the 2^−ΔΔCt^ method. Primers sequences were used as follows:

KRT6, 5′-GGGTTTCAGTGCCAACTCAG-3′ (forward) and

5′-CCAGGCCATACAGACTGCGG-3′ (reverse);

KTR16, 5′-TTCCCCAGCTGCATATAAAGGT-3′ (forward) and

5′-GCAGTTGGCTGAAAGAAGGAAA-3′ (reverse);

MIR31HG, 5′-TCCCAGTTTCAGACCACC-3′ (forward) and

5′-CCAGGCTATGTCTTTCCTCTAT-3′ (reverse);

p65, 5′-GCGAGAGGAGCACAGATACC-3′ (forward) and

5′-AGGGGTTGTTGTTGGTCTGG-3′ (reverse);

GAPDH, 5′-CACATGGCCTCCAAGGAGTAA-3′ (forward) and

5′-TGAGGGTCTCTCTCTTCCTCTTGT-3′ (reverse).

### siRNAs and transfection

The small interfering RNAs (siRNAs) were designed and obtained from Guangzhou RiboBio Co., Ltd (Guangdong, China) and transfected with riboFECT™ CP Transfection Kit (RiboBio) according to the manufacturer’s protocol. Briefly, HaCaT keratinocytes were cultured to 30–50% confluence and then transfected with 50 nM scramble siRNA (siNC) or specific siRNA for 24–96 h.

The sequences of siRNAs were 5′-TCACTCTACACTCTTGATTTT-3′ (siMIR31HG) and 5′-GGATTGAGGAGAAACGTAATT-3′ (sip65).

### CCK8 assay

Cell viability was measured using Cell Counting Kit-8 (CCK8) (Dojindo Molecular Technologies, Inc., Kumamoto, Japan). HaCaT keratinocytes were seeded into 96-well plates, cultured to 30–50% confluenceand then transfected with siRNAs for 24, 48, 72 and 96 h. CCK-8 solution was added and incubated for 2 h at 37 °C. Absorbance at 450 nm was measured using Multiskan Spectrum (ThermoFisher).

### EdU assay

EdU (5-ethynyl-2′-deoxyuridine) incorporation assay was performed using iClick™ EdU Andy Fluor™ 488 Imaging Kit (GeneCopoeia, Guangzhou, China) according to the manufacturer’s protocol. Briefly, HaCaT keratinocytes were seeded into 24-well plates, cultured to 30–50% confluenceand then transfected with siRNAs for 48 h and then incubated with 50 μM EdU for 2 h at 37 °C. Cells were fixed with 4% paraformaldehyde for 15 min, permeabilized with 0.5% Triton X-100 for 20 min, then stained with iClick EdU solution and Hoechst 33342 in dark. Images were captured using Nikon Eclipse Ti-U microscope. EdU incorporation rate was analyzed using Image-pro Plus 6.0 software.

### Flow cytometry analysis of cell cycle

HaCaT keratinocytes were seeded into 6-well plates, cultured to 30–50% confluence and then transfected with siRNAs for 48 h. Cells were harvested, washed with cold phosphate-buffered saline (PBS) and fixed in 70% ethanol at 4 °C overnight. RNA was removed by using RNase A at 37 °C for 30 min. Finally, Cells were stained with propidium iodide (PI) for 30 min at room temperature and analyzed on FACS Aria III flow cytometer (BD Biosciences).

### Statistical analysis

Data were presented as means ± standard errors of three independent experiments. Statistical significance was determined by Student’s t test. A *P* values < 0.05 was considered statistical significant.

## Abbreviations

lncRNAs: long non-coding RNAs
MIR31HG: miR-31 host gene
KRT6: keratin 6
KRT16: keratin 16
siRNA: small interfering RNA
qRT-PCR: quantitative real-time PCR
CCK8: Cell Counting Kit-8
EdU: 5-ethynyl-2′-deoxyuridine
PI: propidium iodide
